# The association of perinatal and clinical factors with outcomes in infants with gastroschisis—a retrospective multicenter study in Finland

**DOI:** 10.1007/s00431-021-03964-w

**Published:** 2021-02-02

**Authors:** Asta Tauriainen, Ulla Sankilampi, Arimatias Raitio, Tuomas Tauriainen, Ilkka Helenius, Kari Vanamo, Anna Hyvärinen

**Affiliations:** 1 Department of Pediatric Surgery, University of Eastern Finland and Kuopio University Hospital, Kuopio, Finland; 2 Department of Pediatrics, Kuopio University Hospital, Kuopio, Finland; 3 Department of Pediatric Surgery and Orthopedics, University of Turku and Turku University Hospital, Turku, Finland; 4 Department of Surgery, Oulu University Hospital, Oulu, Finland; 5 Department of Orthopedics and Traumatology, University of Helsinki and Helsinki University Hospital, Helsinki, Finland; 6 Department of Pediatric Surgery, University of Tampere and Tampere University Hospital, Tampere, Finland

**Keywords:** Gastroschisis, Outcomes, Liver herniation, Central line sepsis

## Abstract

The aim of the present study was to assess the prognostic factors for the outcome of gastroschisis in Finland. A retrospective multicenter study of gastroschisis patients born between 1993 and 2015 in four Finnish university hospitals was undertaken, collecting perinatal, surgical, and clinical data of neonates for uni- and multifactorial modeling analysis. The aim of the present study was to identify risk factors for mortality and the composite adverse outcome (death and/or short bowel syndrome or hospital stay > 60 days). Of the 154 infants with gastroschisis, the overall survival rate was 90.9%. In Cox regression analysis, independent risk factors for mortality included liver herniation, pulmonary hypoplasia, relaparotomy for perforation or necrosis, abdominal compartment syndrome, and central line sepsis. Furthermore, a logistic regression analysis identified central line sepsis, abdominal compartment syndrome, complex gastroschisis, and a younger gestational age as independent predictors of the composite adverse outcome.

*Conclusion*: The risk of death is increased in newborns with gastroschisis who have liver herniation, pulmonary hypoplasia, abdominal compartment syndrome, relaparotomy for perforation or necrosis, or central line–associated sepsis. Special care should be taken to minimize the risk of central line sepsis in the clinical setting.

**Table:** 

**What is known:** • *Gastroschisis is a relatively rare congenital anomaly of the abdominal wall and its incidence is increasing*. • *Complex gastroschisis has been reported to increase risk of mortality and complications*. **What is new:** • *Central line sepsis was found to be independently associated with mortality in gastroschisis patients*. • *Liver herniation was also significantly associated with mortality*.

**What is known:**

• *Gastroschisis is a relatively rare congenital anomaly of the abdominal wall and its incidence is increasing*.

• *Complex gastroschisis has been reported to increase risk of mortality and complications*.

**What is new:**

• *Central line sepsis was found to be independently associated with mortality in gastroschisis patients*.

• *Liver herniation was also significantly associated with mortality*.

## Introduction

Gastroschisis is a congenital defect of the anterior abdominal wall allowing protrusion of the intestines and other abdominal organs out of the abdominal cavity. Prevalence of this condition varies from one to five in 10 000 live births and is increasing in the western countries [1–5]. Gastroschisis is associated with a significant short-term morbidity, including sepsis and gastrointestinal dysfunction [6, 7]. Infants born with gastroschisis frequently require long hospitalization in the neonatal period and sometimes also thereafter [8, 9], but the reported survival rate is over 90% [2, 3, 7, 9, 10].

Approximately 40% of infants with gastroschisis have at least one prolapsed intra-abdominal organ in addition to prolapsed small and/or large bowel. Interestingly, prolapsed organs have been associated with improved outcomes, potentially as a result from a larger fascial defect allowing adequate blood flow and thus reducing bowel necrosis or perforations [11]. However, liver herniation has been associated with a high rate of mortality [12]. According to literature, infants with a complex gastroschisis defined as bowel atresia, perforation, necrosis, or volvulus have an increased risk of mortality, up to 15% [2, 7, 9]. Data on the predictors of outcome in gastroschisis are scarce. Knowing factors associated with unfavorable or favorable outcome would be valuable for prenatal and postnatal counseling of parents, as well as guiding clinicians who treat the patients. Furthermore, some risks of unfavorable outcome of gastroschisis could be avoidable. The aim of the present study was, using a multicenter retrospective setting, to investigate perinatal and clinical factors that are independently associated with death or composite adverse outcome (death, short bowel syndrome, and/or hospital stay > 60 days) in infants with gastroschisis.

## Materials and methods

### Patients

We conducted a retrospective study of neonates born with gastroschisis between the 1 January 1993 and 31 December 2015 and treated in four university hospitals, Tampere, Turku, Kuopio, and Oulu, Finland. The patients were identified in the hospital registers based on the diagnoses (ICD-9 code 756.73, and after the year 1994 ICD-10 code Q79.3). Data on perinatal factors (maternal data, prenatal examinations, initial presentation at birth) and clinical presentation (prolapsed organs, surgical treatment, postoperative treatment, complications, and short-term outcomes) were collected from the patient records and nationwide registers (the Finnish Medical Birth Register and the Finnish Register of Congenital Malformations maintained by the Finnish Institute for Health and Welfare, THL). The study was carried out according to the Finnish national and European Union legislation and guidelines. The institutional review boards of the university hospitals and the Finnish Institution for Health and Welfare accepted the study (THL/206/5.05.00/2017). The need for patients’ written consent was deemed unnecessary by the institutional review boards as we did not contact the families to conduct this retrospective study. Follow-up was assessed using the data provided by THL. Biases were avoided by including a complete cohort of patients. The STROBE cohort reporting guidelines were used in the manuscript [13]. The work described has been carried out in accordance with The Code of Ethics of the World Medical Association (Declaration of Helsinki) for experiments involving humans.

The goal of surgical care was visceral reposition and primary fascial closure in the index operation. If primary closure was impossible, secondary closure with silos was utilized. Patients with too large a defect for closure were treated with a patch. Atresia was treated in a number of ways in the initial operation. Eleven cases of bowel atresia were present in the study; two were missed in the primary operation; three were treated with resection and immediate anastomosis without an ostomy; one with resection, anastomosis, and an ostomy; one with resection and an ostomy; and two with ostomy alone. Moreover, two patients had missing information on the method of atresia treatment.

### Definitions

The birth weight Z scores were calculated using the contemporary Finnish birth size reference [14]. Small for gestational age (SGA) was defined as birth weight below 2 standard deviations (SD) [15]. Patients born before the 37th gestation week were defined as premature. Organ prolapse was defined as any intra-abdominal organ, in addition to bowel, fully or partially protruding through the fascial defect at birth. Patients with associated bowel atresia, bowel perforation, bowel necrosis, or volvulus were defined to have a complex gastroschisis, as suggested by Molik and Abdullah [16, 17]. Septic infections associated with the peripherally or centrally inserted central lines were defined as positive blood and/or catheter tip cultures combined to clinically septic presentation registered in patient records. Silo closure of the gastroschisis was defined as staged closure using commercial spring-loaded silo or sutured silastic silo or sutured Gore-Tex silo. Direct closure was defined as sutured skin and/or fascia with or without fascial patch. Silo complication was defined as detached silo or silo-related hemodynamic complication. Time to enteral nutrition was defined as the number of days since birth before any per oral nutrition was started. Enteral tube feeding is also considered as per oral nutrition. Length of parenteral nutrition was defined as the number of days since birth until intravenous glucose, amino acids, and/or lipid infusions were stopped.

### Outcomes

The primary outcomes in this study were death and the composite adverse outcome of death, short bowel syndrome, or the duration of hospital stay of more than 60 days.

### Statistical analyses

Statistical analyses were performed using SPSS software (version 25.0, IBM Corporation, New York, USA). No attempt to replace missing values was made. Categorical variables were reported as counts and percentages. Continuous variables were reported as median and interquartile range in addition to range. First, the potential predictive factors for death or composite adverse outcome (death and/or prolonged hospitalization or short bowel syndrome) were assessed using Chi-square and Fischer’s exact tests for categorical variables and Mann-Whitney U test (variables without normal distribution) and independent samples *t* test (variables with normal distribution) for continuous data. Normality of the variables were assessed using the Shapiro-Wilk test. A Cox logistical regression analysis was undertaken to identify independent risk factors for mortality. Variables with a *p* value < 0.1 in the univariate analyses were included in this model. These were the gestational age at birth, Apgar score at 1 min of age, a staged closure with silo, liver herniation, pulmonary hypoplasia, silo complications, relaparotomy for bowel perforation or necrosis, abdominal compartment syndrome, and central line sepsis.

Due to our relatively small patient population and few deceased patients, the list of significant variables (*p* < 0.05) in univariate analysis is short: silo closure, defect size in cm, liver herniation, relaparotomy for perforation/necrosis, central line sepsis, and time of parenteral nutrition. Adding all these aforementioned factors to the Cox regression model would induce multicollinearity, which decreases the reliability of the results. Avoiding multicollinearity reduces the number of variables in the multivariate model. For this reason, a *p* value < 0.1 was chosen as a compromise in the Cox regression model. This enables the inclusion of previously known predictors of mortality to the model, such as pulmonary hypoplasia, abdominal compartment syndrome, patient age, and Apgar score.

Variables with a *p* value < 0.05 in the univariate analyses were included in the logistic regression assessing independent predictors for the composite adverse outcome. The model utilized the following variables: silo complications, complex gastroschisis, gestational age, relaparotomy for perforation or necrosis, abdominal compartment syndrome, Apgar score at 1 min of age, and central line sepsis. Multicollinearity was avoided by excluding related factors in both models. All statistical tests were performed as two-tailed with a *p* value < 0.05 representing statistical significance.

## Results

A total of 155 newborns with gastroschisis born between 1 January 1993 and 31 December 2015 were identified. One neonate with missing patient records was excluded. Maternal and neonatal characteristics of the 154 patients are summarized in Table 1. There were 86 (55.8%) male and 68 female infants. Gastroschisis was diagnosed prenatally in 130 (84.4%) cases. Thirteen (7.8%) patients were born in a central hospital, requiring a transfer to a university hospital postnatally. The median gestational age at birth was 36.9 weeks and the median birth weight was 2470 g. A third (31.2%) of infants were born vaginally. Primary repair with direct closure using a skin or fascia was performed in 92 (59.7%) cases. The median fascial defect size was 3.0 cm (the data were available in 59 [38.3%] cases). Twenty-one (13.6%) patients had a complex gastroschisis. The number of surviving patients in the present study was 140 (90.9%) during the follow-up time (mean 11 years, range from 1 day to 26 years) and 14 had died. Seven (4.5%) patients died already during the neonatal period (first 28 days after birth), and altogether 13 (8.4%) deceased within the first year.

**Table 1:** Maternal and neonatal characteristics of the study subjects according to outcome

Variables	All (*n* = 154)	Survivors (*n* = 140)	Deceased (*n* = 14)	*P* value	Favorable outcome (*n* = 124)	Adverse outcome (*n* = 30)	*P* value
Maternal age (years)	23.5 ± 6.0 [16.0–40.0]	23.5 ± 6.0 [16.0–40.0]	23.5 ± 4.5 [17.0–32.0]	0.830	24.0 ± 7.0 [16.0–40.0]	23.0 ± 4.0 [17.0–35.0]	0.982
Prenatal diagnose	130 (84.4)	121 (89.0)	9 (75.0)	0.164	109 (89.3)	21 (80.8)	0.317
Vaginal birth	48 (31.2)	42 (30.0)	6 (42.9)	0.368	36 (29.0)	12 (40.0)	0.245
Gestational age at birth (weeks)	36.9 ± 2.4 [29.3–40.3]	36.9 ± 2.3 [29.3–40.3]	35.8 ± 2.1 [31.4–38.8]	0.065	36.9 ± 2.1 [30.4–40.3]	36.4 ± 2.5 [29.3–39.9]	0.014
Prematurity	82 (53.2)	72 (51.4)	10 (71.4)	0.153	62 (50.0)	20 (66.7)	0.101
Birth weight (grams)	2470 ± 710 [1000.0–3960.0]	2480 ± 680 [1000.0–3960.0]	2430 ±1090 [1660.0–3500.0]	0.625	2560 ± 690 [1380.0–4960.0]	2370 ± 660 [1000.0–3640.0]	0.151
Low birth weight < 2500 g	80 (51.9)	72 (51.4)	8 (57.1)	0.683	60 (48.4)	20 (66.7)	0.072
SGA	56 (36.4)	52 (37.1)	4 (28.6)	0.525	47 (37.9)	9 (30.0)	0.419
Male	86 (55.8)	76 (54.3)	10 (71.4)	0.218	71 (57.3)	15 (50.0)	0.473
Gemini	6 (3.9)	6 (4.3)	0 (0)	1.000	6 (4.8)	0 (0)	0.598
Apgar 1 min	8.0 ± 2.0 [1.0–10.0]	9.0 ± 2.0 [1.0–10.0]	8.0 ± 4.3 [1.0–9.0]	0.092	9.0 ± 2.0 [1.0–10.0]	8.0 ± 2.5 [1.0–9.0]	0.014
Apgar 5 min	9.0 ± 1.0 [1.0–10.0]	9.0 ± 1.0 [1.0–10.0]	8.0 ± 3.0 [3.0–9.0]	0.169	9.0 ± 1.0 [1.0–10.0]	9.0 ± 1.0 [3.0–10.0]	0.076
Hospital transfer after birth	12 (7.8)	10 (7.2)	2 (14.3)	0.302	9 (7.3)	3 (10.3)	0.700
Pulmonary hypoplasia	1 (0.6)	0 (0)	1 (7.7)	0.086	0 (0)	1 (3.4)	0.192
Bowel atresia	11 (7.1)	10 (7.2)	1 (7.7)	1.000	3 (2.4)	8 (27.6)	0.000

Categorical variables are reported as counts and percentages. Continuous variables are reported as median and interquartile range with range reported between brackets. Adverse outcome: death and/or short bowel syndrome and a hospital stay longer than 60 days. SGA, small for gestational age

We performed univariate analyses to identify potential risk factors for death and for a composite adverse outcome. Perioperative factors associated with death or adverse composite outcome are presented in Table 2. There were statistically significant differences in the patient population with adverse outcome compared with favorable outcome group. Lower gestational age, bowel atresia, lower 1-min Apgar score, signs of bowel ischemia at birth, and complex gastroschisis were all significantly more common among patients in the adverse outcome group. Furthermore, large size of fascial defect was associated with better survival: 3.0 cm in the survivors versus 2.0 cm among the deceased, *p* = 0.017. Patients with gastroschisis who had liver herniation were less likely to survive compared to patients who did not have liver prolapse: 5 (3.6%) cases among survivors versus 4 (28.6%) cases in deceased group, *p* = 0.004. However, it did not reach a statistical significance in composite adverse outcome patients 13.3% versus 4.0% compared to patients with favorable outcome, *p* = 0.073.

**Table 2:** Perioperative variables in 154 gastroschisis patients classified according to outcome

Variables	All (*n* = 154)	Survivors (*n* = 140)	Deceased (*n* = 14)	*P* value	Favorable outcome (*n* = 124)	Adverse outcome (*n* = 30)	*P* value
Days to fascial closure	7.0 ± 6.0 [0.0–26.0]	7.0 ± 7.3 [0.0–26.0]	4.0 ± 3.5 [1.0–8.0]	0.095	7.0 ± 6.0 [1.0–26.0]	7.0 ± 7.8 [0.0–13.0]	0.610
Silo closure	60 (39.0)	51 (36.4)	9 (64.3)	0.042	44 (35.5)	16 (53.3)	0.072
Direct closure	92 (59.7)	87 (62.1)	5 (35.7)	0.055	79 (63.7)	13 (43.3)	0.041
Bowel perforation noticed at birth	3 (1.9)	3 (2.2)	0 (0)	1.000	1 (0.8)	2 (6.9)	0.093
Signs of bowel ischemia at birth	15 (9.7)	13 (9.5)	2 (18.2)	0.308	6 (4.9)	9 (34.6)	0.000
Defect size in cm *α	3.0 ± 2.0 [1.5–10.0]	3.0 ± 1.7 [1.5–10.0]	2.0 ± 0.0 [1.5–2.0]	0.017	3.0 ± 1.6 [1.5–7.0]	2.5 ± 2.5 [1.5–10.0]	0.423
Complex gastroschisis	21 (13.6)	18 (12.9)	3 (21.4)	0.409	8 (6.5)	13 (43.3)	0.000
Stomach herniation	60 (39.0)	54 (38.6)	6 (42.9)	0.754	50 (40.3)	10 (33.3)	0.481
Liver herniation	9 (5.8)	5 (3.6)	4 (28.6)	0.004	5 (4.0)	4 (13.3)	0.073
Small bowel herniation	151 (98.1)	137 (97.9)	14 (100.0)	1.000	121 (97.6)	30 (100.0)	1.000
Large bowel herniation	132 (85.7)	119 (85.0)	13 (92.9)	0.694	106 (85.5)	26 (86.7)	1.000
Spleen herniation	1 (0.6)	1 (0.7)	0 (0)	1.000	1 (0.8)	0 (0)	1.000
Bladder herniation	12 (7.8)	10 (7.1)	2 (14.3)	0.299	10 (8.1)	2 (6.7)	1.000
Reproductive organ herniation	12 (7.8)	12 (8.6)	0 (0)	0.603	12 (9.7)	0 (0)	0.125
Gallbladder herniation	5 (3.2)	4 (2.9)	1 (7.1)	0.383	4 (3.2)	1 (3.3)	1.000
Appendix vermiformis herniation	2 (1.3)	2 (1.4)	0 (0)	1.000	2 (1.6)	0 (0)	1.000
Pancreas herniation	1 (0.6)	1 (0.7)	0 (0)	1.000	1 (0.8)	0 (0)	1.000

Categorical variables are reported as counts and percentages. Continuous variables are reported as median and interquartile range with range reported between brackets. Adverse outcome: death and/or short bowel syndrome and a hospital stay longer than 60 days. *Missing data in 61.7%. α represents a variable without normal distribution. Complex gastroschisis: bowel atresia, bowel perforation, bowel necrosis, or volvulus

The clinical findings and outcomes are presented in Table 3. In the univariate model, a relaparotomy for perforation or necrosis was a risk factor for mortality. It was found significantly more often among those who died, 4 (30.8%) cases in deceased versus 4 (2.9%) cases in surviving patients (*p* = 0.002). Central line sepsis was also more common in those who did not survive (*p* = 0.011). A total of 20 (13%) patients with central line sepsis were identified, 15 (10.9%) among survivors and 5 (41.7%) in deceased group. The rate of central line sepsis in the present study was 22.2%, 21.2%, 10.2%, and 0.0% in each center, respectively. The surviving patients had significantly longer median parenteral nutrition in days, in a univariate analysis. Relaparotomy for perforation or necrosis, relaparotomy for obstruction, silo complications, abdominal compartment, necrotizing enterocolitis, short bowel syndrome, central line sepsis, length of parenteral nutrition, and time to enteral nutrition were all more common in patients with adverse outcome in univariate analysis.

**Table 3:** Clinical features in 154 newborns with gastroschisis

Variables	Overall series (*n* = 154)	Survivors (*n* = 140)	Deceased (*n* = 14)	*P* value	Favorable outcome (*n* = 124)	Adverse outcome (*n* = 30)	*P* value
Relaparotomy for perforation/necrosis	8 (5.2)	4 (2.9)	4 (30.8)	0.002	3 (2.4)	5 (17.2)	0.007
Relaparotomy for obstruction	14 (9.2)	12 (8.6)	2 (15.4)	0.338	4 (3.2)	10 (34.5)	0.000
Silo complications	5 (3.3)	3 (2.1)	2 (15.4)	0.058	2 (1.6)	3 (10.3)	0.047
Abdominal compartment syndrome	5 (3.3)	3 (2.1)	2 (15.4)	0.058	2 (1.6)	3 (10.3)	0.047
Necrotizing enterocolitis	4 (2.6)	3 (2.1)	1 (7.7)	0.302	1 (0.8)	3 (10.3)	0.022
Short bowel syndrome	7 (4.6)	6 (4.3)	1 (7.7)	0.470	0 (0)	7 (24.1)	0.000
Central line sepsis	20 (13.0)	15 (10.9)	5 (41.7)	0.011	10 (8.2)	10 (37.0)	0.000
Mechanical ventilator time (days)	4.0 ± 6.0 [0.0–83.0]	3.5 ± 5.0 [0.0–36.0]	7.0 ± 9.0 [1.0–83.0]	0.339	4.0 ± 4.8 [0.0–36.0]	3.0 ± 11.0 [1.0–83.0]	0.052
Duration of parenteral nutrition (days) α	21.0 ± 16.0 [1.0–1869.0]	21.0 ± 16.0 [5.0–1869.0]	7.0 ± 25.0 [1.0–35.0]	0.014	19.0 ± 13.0 [5.0–48.0]	30.0 ± 37.0 [1.0–1869.0]	0.002
Duration to enteral nutrition (days)	7.0 ± 7.3 [1.0–39.0]	7.0 ± 6.5 [1.0–39.0]	9.0 ± 10.5 [3.0–18.0]	0.598	6.0 ± 5.0 [2.0–38.0]	9.0 ± 11.0 [1.0–39.0]	0.005
Length of stay (days) α	27.0 ± 18.5 [1.0–372.0]	27.0 ± 17.0 [7.0–372.0]	22.0 ± 59.8 [1.0–163.0]	0.381	25.0 ± 12.8 [7.0–59.0]	39.0 ± 53.5 [1.0–372.0]	0.000

Categorical variables are reported as counts and percentages. Continuous variables are reported as median and interquartile range with range reported between brackets. α represents a variable without normal distribution. Adverse outcome: death and/or short bowel syndrome and a hospital stay longer than 60 days

The factors with a *p* < 0.100 were included in the Cox regression analysis model to determine independent risk factors for mortality (Table 4). The factors that were associated with risk of death in gastroschisis were relaparotomy for perforation or necrosis, central line sepsis, liver herniation, abdominal compartment syndrome, and pulmonary hypoplasia. A logistic regression analysis (Hosmer-Lemesow’s test *p* = 0.736 and area under the curve in ROC analysis of 0.887) including the factors that were significant in the univariate model (*p* < 0.05) identified significant predictors for adverse composite outcome. These were central line sepsis, abdominal compartment syndrome, complex gastroschisis, and a younger gestational age.

**Table 4:** Factors associated with death in Cox regression model (A) or with the composite adverse outcome of death and/or prolonged hospitalization or short bowel syndrome in a logistic regression analysis (B)

A. Independent predictors of mortality	Hazard ratio	95% confidence interval	*P* value
Gestational age	1.001	0.613–1.636	0.996
Apgar 1 min	1.057	0.747–1.496	0.753
Silo closure	0.841	0.163–4.353	0.837
Silo complications	12.977	0.906–185.921	0.059
Relaparotomy for perforation or necrosis	8.221	1.809–37.353	0.006
Central line sepsis	8.903	1.883–42.106	0.006
Liver herniation	8.659	1.017–73.723	0.048
Abdominal compartment	9.573	1.184–77.376	0.034
Pulmonary hypoplasia	27.796	1.136–680.241	0.042
B. Independent predictors of composite adverse outcome	Odds ratio	95% confidence interval	*P* value
Central line sepsis	14.215	3.604–56.069	0.000
Abdominal compartment syndrome	17.614	2.146–144.599	0.008
Silo complications	12.157	0.949–155.656	0.055
Relaparotomy for perforation or necrosis	3.989	0.609–26.147	0.149
Complex gastroschisis	14.289	3.863–53.218	0.000
Gestational age	0.722	0.539–0.968	0.029
Apgar 1 min	0.907	0.696–1.181	0.468

## Discussion

A few independent risk factors for mortality among gastroschisis patients have been reported previously in the literature. Sepsis, birth weight < 2500 g, an additional congenital anomaly, and gestational age < 34 weeks were identified by Fullerton et al. (2017) [10] and Brebner et al. (2020) [3]. In the present study, a central line–associated sepsis was an independent predictor for death. Contradictory results regarding septicemia and the risk of death are presented in the literature. Fullerton and colleagues (2017) found that sepsis was the sole independent predictor of mortality in their multivariate analysis of 4420 gastroschisis patients [10]. However, Snyder et al. (2020) did not find any association between central venous cannula sepsis and mortality in their group of 2032 patients with gastroschisis. Their study was based on register databases and the incidence of central line–associated blood stream infections was 3.99% [18], which is much lower than the 13% in the present data. In a study by Roberts and Gollow (1990), a 10.7% incidence of central venous catheter sepsis proven by blood and catheter tip cultures, in surgical neonates, was reported [19]. A comparison of the incidence of central line–associated sepsis is difficult due to varying definition criteria and study designs, since only a few studies have been published regarding central line infections in patients with gastroschisis. Unfortunately, the rate of central line sepsis in Finnish newborns in general is not known. In the years 1999–2019, the prevalence of blood culture–positive septicemias in Finnish newborns was 0.7 per 1000 days treated in hospital [20]. The protocols regarding line care in our participating centers were similar; in the setting of elevated inflammatory parameters such as leucocytosis and CRP and in the presence of unalarming abdominal status, the source of infection was searched elsewhere, i.e., central and peripheral lines. These lines were removed if suspected of being infected.

Abdominal compartment syndrome is a serious condition with varying presentations. High intra-abdominal pressure can lead to vascular compromise and bowel ischemia resulting in complications. Oliguria/anuria, peritonism, abdominal distension, hemodynamic or respiratory insufficiency, increased central venous pressure, and organ dysfunction including ileus can be a clinical symptoms warning of high abdominal pressure [21]. The literature suggests that abdominal compartment syndrome could be avoided by careful monitoring of intra-abdominal pressures [22], and a cutoff point of 20 mmHg has been suggested. The risk of abdominal compartment syndrome has been reported to be significantly more common among patients undergoing direct closure of congenital abdominal wall defect [23]. Thus, it can be speculated that orderly treatment with silo could be a more favorable option to an aggressive attempt to fascial closure, which could lead to abdominal compartment syndrome.

McClellan et al. (2011) reported 7 (6%) patients with liver herniation [12], which is comparable to our finding of 5.8% (9 cases). In their univariate analysis, liver herniation was significantly associated with mortality. The present study confirmed these findings. The detrimental effects of liver herniation might be due to the organ’s central location and importance for the surrounding vascular and soft tissue structures. Too fast reduction of a liver prolapse could alter vital functions such as hepatic and portal blood flow, biliary flow, and metabolic as well as protein synthesis functions. Koehler et al. [11] evaluated the importance of organ prolapse in their study. However, their multivariate regression model did not find any statistically significant differences between patients with and without organ prolapse. In their univariate analysis, patients with organ prolapse tolerated enteral feeds earlier, were weaned off total parenteral nutrition earlier, and had a shorter hospital stay [11]. In the present study, we reported 76 (49.4%) patients with prolapsed organs which was slightly higher than the 40.6% observed by Koehler et al. [11]. Similarly, the stomach was the most common organ protruding outside of the abdominal cavity in our study 39.0% versus 26.3% in their study [11].

Another independent predictor of mortality in our model was pulmonary hypoplasia, which has also been previously reported to predict mortality [9]. Pulmonary hypoplasia is commonly caused by other associated anomalies, especially those involving the thoracic cavity or diaphragm, in addition to disruption in normal intrauterine conditions [24] leading to decreased pulmonary development. Moreover, pulmonary hypoplasia as independent predictor of death is likely to be associated with its severity and affects patients in a case-by-case fashion, without many chances for the physician to intervene. However, it is important to acknowledge this risk factor as early as possible in order to prepare for the situation.

According to our multivariate model in Table 4, a great attention to detail should be pursued at the primary operation in order to minimize any risk that could lead to relaparotomy for necrosis or perforation. Relaparotomy for obstruction was not associated with mortality in our univariate analysis, which might be explained by the lack of peritonitis in these patients.

In the present study, complex gastroschisis did not have a statistically significant association with mortality, whereas it significantly increased the risk of a composite adverse outcome endpoint. Previously, complex gastroschisis has been shown to be a predictor mortality [7] and morbidity [25]. Moreover, Ghionzoli et al. (2012) reported that intestinal atresia was significantly associated with prolonged intravenous nutrition; however, it did not increase mortality [26].

Previously known risk factors in the literature for a prolonged hospitalization include bowel resection, sepsis, presence of other congenital anomalies, necrotizing enterocolitis, and a small weight for gestational age (SGA) [10]. Brebner et al. [3] reported that a birth weight < 2500 g was an independent predictor for poor outcome in their sizeable analysis of 4803 gastroschisis patients [3]. The independent predictors for poor outcome in our analysis were central line sepsis, abdominal compartment syndrome, complex gastroschisis, and younger gestational age. The aforementioned factors can be seen as the main determinants of prolonged hospitalization, which is shown in the results of Table 3. In more detail, we speculate that central line sepsis and younger gestational age might increase the need for a longer time in neonatal care, whereas abdominal compartment syndrome and complex gastroschisis could lead to further surgical problems resulting in a short bowel syndrome.

The only modifiable risk factors for mortality and composite adverse outcome found in the present study were the central line–associated sepsis and abdominal compartment syndrome. Indeed, neonates with gastroschisis often require prolonged parenteral nutrition necessitating central line insertion. It is of utmost importance to carefully inspect any signs of central line complications to avoid bacterial colonization or septicemia. Prompt replacement or removal of these lines is mandatory when infection is suspected. However, factors such as the need for relaparotomy due to intestinal perforation or necrosis and abdominal compartment syndrome may also be influenced by the management details and should be kept in mind when choosing the treatment. Indeed, it could be speculated that the improvement in the quality of surgical and intensive care of newborns with gastroschisis during the last two decades has led to the better survival of complex patients. Since patients with complex gastroschisis are no longer at such a high risk for death, as the results of the present study suggests, it is reasonable to assume that this is at the expense of increased morbidity.

Some important limitations must be acknowledged regarding the present study. This is a retrospective study with a relatively small sample size. The patients were treated in four different hospitals with varying surgical treatment protocols. The patients were also operated on by a number of different surgeons and the approach to treating atresia varied. However, only one (1/11, 9.1%) patient with atresia died at the age of 68 days, and in this case the treatment method was resection and immediate anastomosis. This patient underwent five additional abdominal operations in addition to the primary procedure and suffered from postoperative volvulus. Eight (72.7%) patients with atresia were classified as having the composite adverse outcome. In this light, the treatment method of atresia might not have had a significant effect on our results. The hospitals in the present study have different protocols whether to use a tunneled central line or peripherally inserted central cannula; however, the lines were replaced or removed in all centers if an infection was recognized.

## Conclusions

Based on the present study, the risk of death in gastroschisis is increased in newborns who present with liver herniation, pulmonary hypoplasia, abdominal compartment syndrome, relaparotomy for perforation or necrosis, and central line–associated sepsis. Furthermore, central line–associated sepsis, abdominal compartment syndrome, complex gastroschisis, and young gestational age increase the risk of composite adverse outcome. While some of the risk factors for mortality and unfavorable outcome are unavoidable, a meticulous and timely neonatal and surgical care with precise attention to detail enables the treatment among many of these high-risk patients with a possibility of preventing adverse outcomes and improving survival.

## Data Availability

The data and our research material are not available for readers.

## Abbreviations

ICD-10: International Classification of Diseases No. 10
SGA: Small for gestational age
SD: Standard deviation
THL: The Finnish Institution for Health and Welfare
